# *SOD* mRNA and MDA Expression in Rectus Femoris Muscle of Rats with Different Eccentric Exercise Programs and Time Points

**DOI:** 10.1371/journal.pone.0073634

**Published:** 2013-09-13

**Authors:** Heng Zhao, Jiani Liu, Shinong Pan, Yingwei Sun, Qi Li, Fei Li, Li Ma, Qiyong Guo

**Affiliations:** 1 Department of Radiology, Shengjing Hospital of China Medical University, Shenyang, China; 2 Central Laboratory, Shengjing Hospital of China Medical University, Shenyang, China; Universidad Europea de Madrid, Spain

## Abstract

**Purpose:**

Although superoxide dismutase (SOD) and malondialdehyde (MDA) affect Delayed Onset Muscle Soreness (DOMS), their effects are unclear in rectus femoris muscles (RFM) of rats with different eccentric exercise programs and time points. The purpose of this study is to investigate the effects of the various eccentric exercise programs at different time points on the *SOD* mRNA expression and MDA using rat as the animal model.

**Methods:**

248 male rats were randomly divided into 4 groups: control group (CTL, *n* = 8), once-only exercise group (OEG, *n* = 80), continuous exercise group (CEG, *n* = 80), and intermittent exercise group (IEG, *n = *80). Each exercise group was divided into 10 subgroups that exercised 0.5 h, 6 h, 12 h, 24 h, 48 h, 72 h, 96 h, 120 h, 144 h, or 168 h. Rats were sacrificed and their *SOD* mRNA expression, and MDA concentrations of skeletal muscle tissue were measured.

**Results:**

The specimen in all eccentric exercise programs showed increased RFM *SOD1* mRNA expression levels at 0.5 h (*P<*0.05), and decreased RFM *SOD3* mRNA expression at 0.5 h (*P<*0.05). The continuous eccentric exercise (CE) significantly enhanced muscle *SOD2* mRNA level at 0.5 h (*P<*0.05). After once-only eccentric exercise (OE), *SOD1*, *SOD2*, and *SOD3* mRNA expression significantly increased at 96 h, whereas MDA concentrations decreased at 96 h. After CE, the correlation coefficients of *SOD1*, *SOD2*, *SOD3* mRNA expression levels and MDA concentrations were −0.814, −0.763, −0.845 (all *P<*0.05) at 12 h.

**Conclusion:**

Regular eccentric exercise, especially CE could enhance *SOD1* and *SOD2* mRNA expression in acute stage and the *SOD2* mRNA expression correlates to MDA concentration *in vivo*, which may improve the oxidative adaption ability of skeletal muscles.

## Introduction

Delayed-onset muscle soreness (DOMS) is the local pain and discomfort that develops 12–48 h after intensive and/or unusual eccentric exercise muscle action [1], [2]. DOMS can decrease skeletal muscle contractile function and cause myodynamia weakness that can seriously affect a competitive athlete's performance [3]. Currently, the mechanism of DOMS is unclear, but the etiology of DOMS is usually attributed to the establishment of an acute-phase inflammatory response that results from metabolic, mechanical, and oxidative stress, eccentric exercise is the main exercise mode that results in DOMS [4]–[7]. Physical exercise can increase systemic oxygen consumption, causing generation of excessive reactive oxygen species (ROS) that elicit oxidative stress reactions in turn. Muscular oxidative stress induced by exercise training is the most obvious of these reactions and can cause muscular oxidative damage [8].

Superoxide dismutase (SOD) is the first enzymatic line of antioxidant and is an important enzyme in the antioxidant system, it could convert O_2_^−^ to hydrogen peroxide (H_2_O_2_), thereby SOD were regarded as which could protect muscular oxidative stress from exercise effectively [9]. SOD exists in three isoforms, two of which are intracellular: SOD1, which accounts for approximately 90% of total SOD and exists in the cytoplasm and combines with copper or zinc to form a dimer; and SOD2, an inducible mitochondrial enzyme that combines with manganese to form a tetramer. SOD3 exists in the extracellular space and combines with copper or zinc to form a tetramer [10]–[13]. Malondialdehyde (MDA) is a peroxidation product of lipids, and indirectly reflects the degree of ROS on membrane lipid peroxidation [14].

Studies of the role of SOD in DOMS have focused mainly on protein content and activity of SOD isoenzymes [15]–[17]. Some studies suggest that concentric endurance exercise could improve the protection of skeletal muscle from oxidative stress [18]. Increased SOD1 and SOD2 activity was observed in rat soleus after endurance exercise but no change was seen in expression of *SOD1* and *SOD2* mRNA [19], [20]. Although the content and activity of SOD is an important reflection of the antioxidant stress capacity of the body, SOD mRNA expressions also contributed to the antioxidant system. The studies of SOD knockout mice showed that lack of SOD lead to increased oxidative stress and damage in organs [21]–[24]. Overexpression of SOD in transgenic rats is regarded as an effective way to improve cellular defenses against ROS toxicity [10], [25], [26]. These conclusions suggested that SOD gene expressions contributed to the system of antioxidant. Furthermore, the mRNA expressions of enzyme is an adaption ability of skeletal muscle gene after exercise, and the mRNA expressions of SOD reflect the ability of ROS elimination, so the *SOD* mRNA expressions are regarded as one of index in organs adapting to oxidative stress during training [27]–[29].

There always is unclear about *SOD* mRNA expression and MDA in DOMS in previous studies, Hitomi suggested that increased *SOD3* mRNA expression was observed in mouse gastrocnemius after acute uphill treadmill exercise, but with no change in *SOD1* or *SOD2* mRNA expression [28]. In previous studies, the correlation and interaction of SOD and MDA concentration were inconsistent, with some studies indicating MDA concentration to be negatively correlated with SOD activity [14], [15], [30], [31], and others indicating MDA concentration to be potentially associated with *SOD2* mRNA expression [32]. We suggest that this discrepancy might be related to different exercise programs and observation time points, and presume that the different exercise programs and observation time points could induce different *SOD* mRNA expressions and MDA.

Three eccentric exercise models were established in Sprague-Dawley (SD) rats: once-only eccentric exercise (OE), intermittent eccentric exercise (IE), and continuous eccentric exercise (CE). Expression of *SOD* mRNA and concentration of MDA in the rectus femoris muscle (RFM) were determined separately at different time points in SD rats subjected to different eccentric exercise programs using real-time polymerase chain reaction (PCR) and enzyme-linked immunosorbent assay (ELISA), to ascertain the effects of different eccentric exercise programs, different time points, and the interaction between these variables on expression of *SOD* isoenzyme mRNA and MDA concentration in the RFM of SD rats.

## Materials and Methods

### Animals and experimental groups

**Figure 1:**
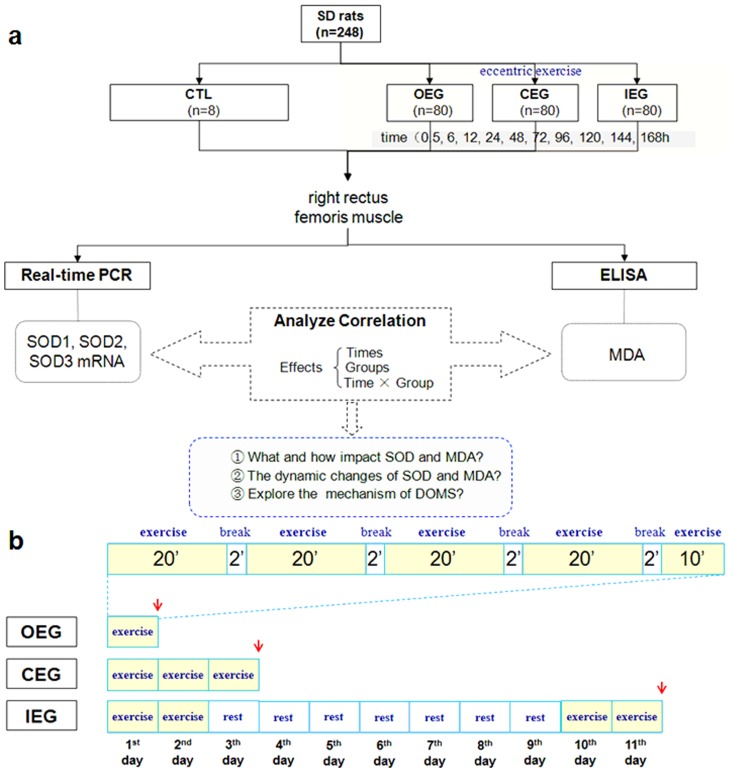
Flow chart (a) and exercise programs (b) of the study. Red arrows indicate the last exercises of 3 exercise programs.

The animal studies and experimental procedures were approved by the Ethics Committee of Shengjing Hospital. A schematic diagram of the experiment is shown in Fig. 1a. The 248 male SD rats (specific-pathogen-free grade; body weight: 197±10 g; age: 6.9±0.9 weeks) used in the study were from the Animal Center of the Shengjing Hospital of China Medical University. All rats were kept in individual cage with standard food and water ad libitum in proper environmental conditions. The rats were randomly divided into four groups as follows: control group (CTL; *n* = 8); once-only exercise group (OEG; *n* = 80) which exercised only once; continuous exercise group (CEG; *n* = 80) which exercised once every 24 h for three times continuously; and intermittent exercise group (IEG; *n* = 80) which exercised once every 24 h for two times continuously and then two more times continuously after a 7-day break (Fig. 1b). Each of the 3 exercise groups was further divided into 10 subgroups and the rats were sacrificed according to the time of their last exercise: 0.5 h, 6 h, 12 h, 24 h, 48 h, 72 h, 96 h, 120 h, 144 h, or 168 h for each exercise group (n = 8 for each subgroup).

### Exercise program

All rats in the exercise groups were familiarized with treadmill running by exercising on a motor-driven treadmill for two days (5–10 min/day, 5–10 m/min, at 08:00 AM). Having acclimatized to the treadmill, each rat in the 3 exercise groups was performed treadmill running at a slope of −15°, 5 min adaptation exercise at a speed of 10 m/min, and then 20 min exercise at a speed of 21 m/min with a 2 min break, according to Lima-Cabello et al [33] with modifications. Each bout in the 3 exercise groups exercise for 90 min (Fig. 1b). The stimulation method included weak electrical stimulation, blunt-nib simulation on the rat's back, and photic stimulation. Rats in the control group did not exercise.

### Tissue preparation

The rats in the each time-point post-exercise subgroup of the 3 exercise groups were anesthetized with an intraperitoneal injection of 10% chloral hydrate (0.35 ml/100 g), and sacrificed by decapitation. The right RFM was dissected immediately and divided into two blocks: tissue block No.1 (approximately 20 mg) and tissue block No.2 (approximately 200 mg) were placed in liquid nitrogen and both stored at −80°C. The rats in the control group were anesthetized and sampled in the same way as those in the 3 exercise groups.

After rapid thawing and weighing, muscle samples were manually homogenized on ice with a glass-Teflon homogenizer, in 9 volumes of ice-cold physiological saline. Homogenates were centrifuged for 15 min at 3,000× *g* (Eppendorf 5801R centrifuge, Germany), and the resultant supernatants were collected for the analysis of *SOD* mRNA expression and MDA concentration.

### Real-time PCR analysis

Total RNA was isolated from the frozen tissue block No.1 and reverse transcribed according to the RNAiso Plus instructions (TaKaRa, Otsu, Japan). The volume of the RNA reverse transcription system was 20 µl, and cDNA was synthesized by reverse transcription with 50 pmol oligo dT primer and 100 pmol random hexamers as primers. The reaction conditions were three cycles at 37°C for 15 min and 85°C for 5 s. Thereafter, real-time PCR was performed as reported previously [34].

Glyceraldehyde 3-phosphate dehydrogenase (*GAPDH*) served as the internal reference [35], and the order-specific primer designs for *SOD1*, *SOD2*, *SOD3*, and *GAPDH* were completed by TaKaRa Bio (Table 1). A SYBR Green I Real-time PCR kit was used (TaKaRa Bio). The volume of the reaction system was 20 µl, containing 0.2 µM upstream primer, 0.2 µM downstream primer, 10 µl SYBR Premix Ex Taq, 7.2 µl distilled H_2_O, and 2 µl cDNA. A two-step PCR reaction procedure was used that consisted of pre-denaturation at 95°C for 30 s, a PCR reaction at 95°C for 5 s, and 45 cycles at 60 or 63°C for 20 s. This reaction was performed using a LightCycler Real-time PCR Amplifier (Roche Diagnostics, Penzberg, Germany). LightCycler software, version 3.5 (Roche Molecular Biochemicals, Mannheim, Germany), was used to perform the relative quantification analysis to determine the transcription levels of *SOD1*, *SOD2*, and *SOD3*.

**Table 1:** Order of primer pairs applied in this study.

Gene name	Orders	Annealing temperature
*SOD1*	Forward: 5′AATGTGTCCATTGAAGATCGTGTGA3′	60°C
	Reverse: 5′GCTTCCAGCATTTCCAGTCTTTGTA3′	
*SOD2*	Forward: 5′AGGGCCTGTCCCATGATGTC3′	63°C
	Reverse: 5′AGAAACCCGTTTGCCTCTACTGAA3′	
*SOD3*	Forward: 5′GGGTCTGTCCTGTACTTCACCAGAG3′	60°C
	Reverse: 5′CTGACATGGTCCAGGTGACAGAG3′	
*GAPDH*	Forward: 5′GCACCGTCAAGGCTGAGAAC3′	60°C
	Reverse: 5′ATGGTGGTGAAGACGCCAGT3′	

### MDA determination

MDA determination of skeletal muscle tissue in all rats was quantified by ELISA (Cell Biolabs, USA). A 100-mg sample of muscle tissue No.2 was weighed and added to 0.1 mol/L phosphate buffer (pH 7.4 at 4°C) containing 1.17% KCL to prepare a 20% (w/v) homogenate. The homogenate was centrifuged at 3,000× *g* at 4°C for 20 min and the supernatant fraction was then removed. After dilution and previously described standard ELISA steps [36], determination was performed using a WellScan MK3 spectrophotometer (Labsystems Dragon, Helsinki, Finland) at 450 nm.

### Statistical analysis

SPSS 17.0 software (Surrey, UK) was used for the analysis. All data were presented as mean ± standard deviation. Univariate analysis of variance (ANOVA) was performed to analyze all of the variables. Homogeneity tests were conducted on the data. Comparisons were made between the effects of time and exercise group, and interactions between these effects were then analyzed for each exercise group. The LMATRIX clause of the univariate ANOVA in SPSS was used to analyze the effect of the group and time factors for each subgroup. The correlation between *SOD1*, *SOD2*, and *SOD3* mRNA and MDA concentration was analyzed with the Pearson correlation test. *P*<0.05 was considered statistically significant.

## Results

### Between-subjects effects

Firstly, we examined the existence of time effects. There were significant effects of time in *SOD1*, *SOD2*, and *SOD3* mRNA levels and MDA concentrations were found at the same eccentric exercise programs (all *P*<0.01, Table 2). It indicating that the measurements changes with the time points. Secondly, we examined the existence of group effects. There were significant effects of group in *SOD1*, *SOD2*, and *SOD3* mRNA levels and MDA concentrations were found between different eccentric exercise programs (all *P*<0.001, Table 2). It indicating that the effects of every exercise programs were different. Thirdly, we examined the existence of time × group interactions. There were significant effects of time × group interactions were found for *SOD1*, *SOD2*, and *SOD3* mRNA expression levels and MDA concentrations (all *P*<0.05, Table 2). It indicating that the time effects changed with the group effects.

**Table 2:** *P* value of the effects of time, group, and time × group interaction.

	*SOD1* mRNA	*SOD2* mRNA	*SOD3* mRNA	MDA
Time	0.000	0.001	0.009	0.000
Group	0.000	0.000	0.000	0.000
Interaction	0.000	0.000	0.000	0.026

### Comparison of the effects of time and exercise group on SOD1 mRNA expression

**Figure 2:**
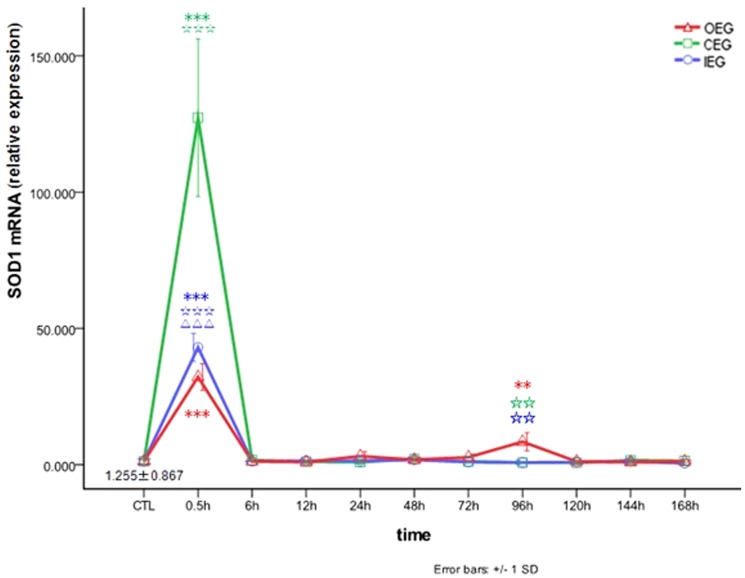
Changes in *SOD1* mRNA in rectus femoris muscle after OE, CE, and IE. **P<0.01, ***P<0.001 vs CTL; ☆☆*P*<0.01, ☆☆☆*P*<0.001 vs OEG. ΔΔΔ *P*<0.001 CEG vs IEG.

We evaluated changes of *SOD1* mRNA expression in the three different experimental procedures and time frame. As shown in Fig. 2, *SOD1* mRNA expression levels in the RFM reached the first peak at 0.5 h after OE program (32.198±4.856-fold increase from CTL, *P*<0.001), returned to normal at 6 h, remained at this level until 72 h, increased again at 96 h (8.447±3.346-fold increase from CTL, *P = *0.007), and then returned to normal at 120–168 h. It indicating that *SOD1* mRNA expression levels were up-regulated at 0.5 h and 96 h after the OE program. After the CE and IE programs, *SOD1* mRNA expression in the RFM reached a peak at 0.5 h (*SOD1* mRNA_CEG_, 127.288±28.909-fold increase from CTL, *P*<0.001; *SOD1* mRNA_IEG_, 43.083±5.103-fold increase from CTL, *P*<0.001), returned to normal at 6 h, and remained at this level until 168 h. These results indicating that *SOD1* mRNA expression were up-regulated levels at 0.5 h after the CE and IE programs.

### Comparison of the effects of time and exercise group on *SOD2* mRNA expression

**Figure 3:**
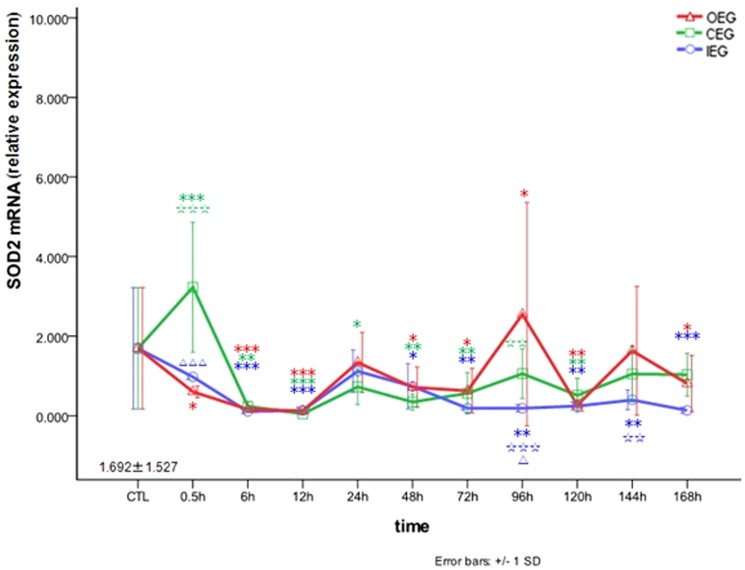
Changes in *SOD2* mRNA in rectus femoris muscle after OE, CE, and IE. * *P*<0.05, ** *P*<0.01, *** *P*<0.001 vs CTL; ☆☆ *P*<0.01, ☆☆☆ *P*<0.001 vs OEG; Δ *P*<0.05, ΔΔΔ *P*<0.001 CEG vs IEG.

We measured changes of *SOD2* mRNA expression in the three different experimental procedures and time frames. As shown in Fig. 3, the expression of *SOD2* mRNA in the RFM increased 0.5 h after CE program (3.231±1.631-fold increase from CTL, *P*<0.001), decreased at 6 to 72 h, returned to normal at 96 h, decreased again at 120 h, and returned to normal at 144–168 h. It indicating that *SOD2* mRNA expression up-regulated at 0.5 h after CE program only. After OE, *SOD2* mRNA expression gradually decreased at 0.5–12 h, returned to normal at 24 h, decreased at 48–72 h, reached a peak at 96 h (2.547±2.810-fold increase from CTL, *P* = 0.048), and decreased after 120 h. After IE, *SOD2* mRNA expression decreased at 6–12 h, returned to normal at 24 h, and decreased again at 48–168 h. These results indicated that up-regulated *SOD2* mRNA expression at 96 h after OE program.

### Comparison of the effects of time and exercise group on *SOD3* mRNA expression

**Figure 4:**
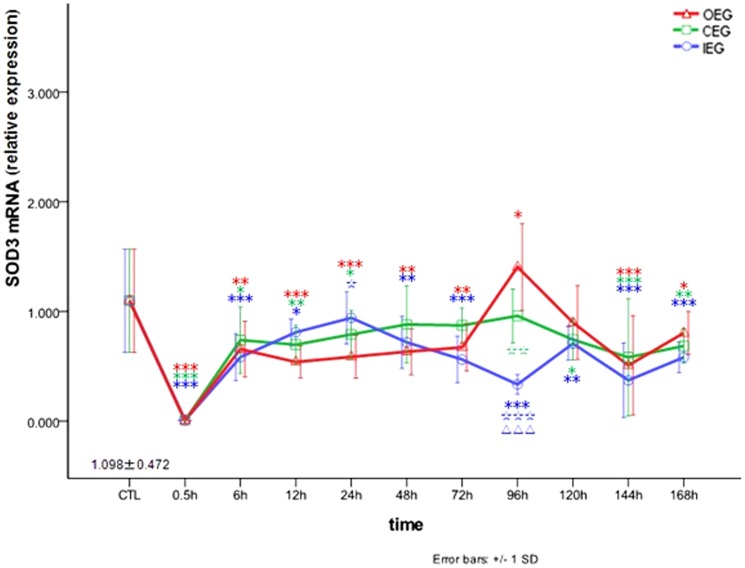
Changes in *SOD3* mRNA in rectus femoris muscle after OE, CE, and IE. * *P*<0.05, ** *P*<0.01, ** *P*<0.001 vs CTL; ☆ *P*<0.05, ☆☆ *P*<0.01, ☆☆☆ *P*<0.001 vs OEG; ΔΔΔ *P*<0.001 CEG vs IEG.

We next evaluated changes of *SOD3* mRNA expression in the three different experimental procedures and time frames. As shown in Fig. 4, *SOD3* mRNA expression in the RFM decreased to a trough at 0.5 h after OE program (0.005±0.001-fold increase, *P*<0.001), stayed at this level until 72 h, increased at 96 h (1.405±0.394-fold increase, *P* = 0.027), returned to normal at 120 h, and decreased again at 144–168 h. It indicating that *SOD3* mRNA expression were up-regulated at 96 h after OE program. After the CE and IE programs, *SOD3* mRNA expression decreased to a trough at 0.5 h in both instances (*SOD3* mRNA_CEG_, 0.007±0.001-fold increase, *P<*0.001; *SOD3* mRNA_IEG_, 0.007±0.004-fold increase, *P*<0.001) and then fluctuated between low and normal levels. It indicating that *SOD3* mRNA expression weren't upregulated after CE and IE programs.

### Comparison of the effects of time and exercise group on MDA concentration

**Figure 5:**
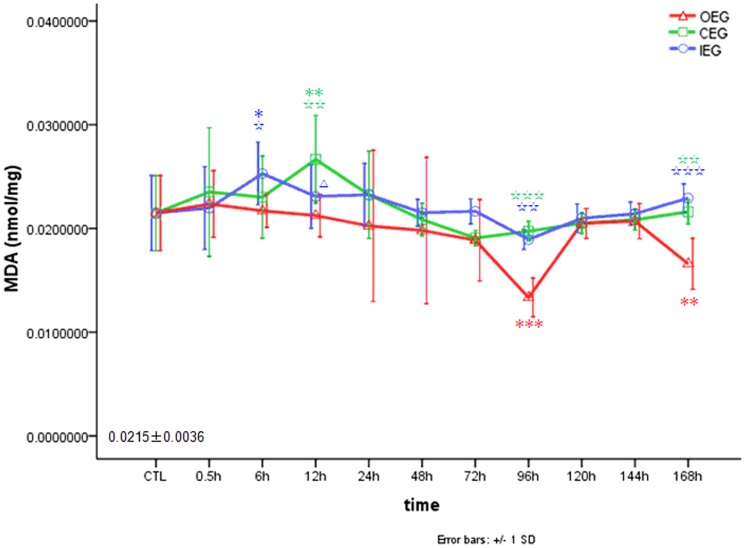
Changes in MDA in rectus femoris muscle after OE, CE, and IE. * *P*<0.05, ** *P*<0.01, *** *P*<0.001 vs CTL (MDA_CTL_ = 0.0215±0.0036 nmol/mg); ☆*P*<0.05, ☆☆*P*<0.01, ☆☆☆*P*<0.001 vs OEG; Δ *P*<0.05 CEG vs IEG.

We measured the MDA levels in RFM tissue, a marker of oxidative stress. As shown in Fig. 5, after OE, MDA concentrations in the RFM remained at normal levels at 0.5–72 h, decreased at 96 h (0.0133±0.0019 nmol/mg tissue, *P*<0.001), increased to normal levels at 120–144 h, and decreased again at 168 h. It indicating that there was down-regulation of MDA concentration at 96 h and 168 h after OE program. After CE, MDA concentrations remained at normal levels at 0.5–6 h, increased at 12 h (0.0267±0.0042 nmol/mg tissue, *P* = 0.001), and then decreased to normal levels at 24–168 h. After IE, MDA concentrations remained at normal levels at 0.5 h, increased at 6 h (0.0253±0.0030 nmol/mg tissue, *P* = 0.018), and returned to normal levels at 12–168 h.

### Correlation coefficients of *SOD1*, *SOD2*, *SOD3* mRNA expressions and MDA concentration

To determine whether the *SOD* mRNA expression correlates to MDA concentration in vivo, we analyzed the correlation between *SOD1*, *SOD2*, *SOD3* mRNA expression and MDA concentration (Table 3). After CE, the correlation coefficients of *SOD1*, *SOD2*, *SOD3* mRNA expression levels and MDA concentrations were −0.814, −0.763, −0.845 (all *P*<0.05) at 12 h, respectively. It indicating that *SOD* mRNA expression negatively correlated with MDA concentrations after CE, especially at 12 h. After OE, the correlation coefficients of *SOD2* mRNA expression levels and MDA concentrations were −0.846, −0.714 (all *P*<0.05) at 48 h and 96 h respectively. It indicating that *SOD2* mRNA expression was correlated with MDA concentrations at more time points.

**Table 3:** Pearson correlation coefficients between *SOD* mRNA expression and MDA concentration.

	Pearson correlation of *SOD1* and MDA			Pearson correlation of *SOD2* and MDA			Pearson correlation of *SOD3* and MDA		
	OEG	CEG	IEG	OEG	CEG	IEG	OEG	CEG	IEG
0.5 h	0.217	0.418	−0.621	0.343	−0.339	−0.352	−0.160	0.411	−0.361
6 h	−0.096	−0.453	0.360	−0.173	−0.474	−0.558	−0.101	0.028	0.562
12 h	−0.023	−0.814*	0.059	0.155	−0.763*	0.079	0.058	−0.845**	−0.245
24 h	0.696	0.247	−0.599	−0.526	0.216	−0.619	0.463	0.094	−0.391
48 h	0.536	−0.341	−0.251	−0.846**	0.051	−0.327	0.476	0.318	−0.319
72 h	0.413	0.031	0.009	−0.294	0.027	0.155	−0.105	−0.023	0.157
96 h	0.031	−0.044	0.350	−0.714*	0.226	0.274	−0.452	−0.083	−0.292
120 h	0.112	0.086	0.505	0.084	0.163	−0.200	0.118	0.063	0.465
144 h	−0.340	−0.109	−0.142	−0.057	−0.106	−0.070	−0.310	0.015	0.141
168 h	0.542	0.144	0.110	−0.282	0.137	0.087	−0.435	0.023	0.084

* *P<*0.05.

** *P<*0.01.

## Discussion

The existence of a free-radical scavenging system in the healthy body means that the body dynamically balances free-radical production and scavenging. The body can generate a large number of oxygen free radicals through various pathways during exercise. Therefore, the inhibition of lipid peroxidation caused by free radicals may have a protective effect on muscle cells [37]. Excessive ROS generation leads to apoptotic and necrotic cell death, but homeostatic and physiological levels of ROS are indispensable in the regulation of diverse cellular processes, including ion channel/transporter function, Ca^2+^ spike production, protein kinase/phosphatase activation, and gene expression [38]. Here, we evaluated the effects of different eccentric exercise programs, different observation time points, and the interaction between these variables on expression of *SOD* isoenzyme mRNA and MDA concentration in the RFM of SD rats.

In the present study, we found that *SOD1* mRNA expression significantly increased by 0.5 h after three types of eccentric exercise, and that *SOD1* mRNA expression is highest after CE program. *SOD1* mRNA expression levels remained normal from 6 h to 144 h after CE and IE programs and remained largely normal for OE program, but increased at 96 h. Therefore, OE, CE, and IE programs were associated with increased *SOD1* mRNA in the RFM during the acute phase. SOD isoenzymes are important antioxidases in the body and indicators of activity of the free-radical-scavenging enzyme systems. Expression of *SOD1* mRNA in the RFM increased rapidly after OE, CE, and IE, indicating that the body increased its protection by increasing *SOD1* expression to improve the ability to scavenge oxygen free radicals and fight against tissue oxidation to protect cells from damage. The redox control of nuclear factor κB (NF-κB) activation is an important signaling pathway, in which skeletal muscles adapt to oxidative stress. The 5′ promoter of *SOD1* contains an NF-κB binding site and the binding level of NF-κB and DNA reach a peak approximately 2 h after acute exercise [39], [40]. Pimenta and Itoh [41], [42] showed that *SOD1* mRNA expression significantly increased after acute contraction of rat skeletal muscles, which supported our data of 0.5 h after OE, CE, and IE. Therefore, we suggest that a common feature of the RFM in SD rats after OE, CE, and IE is a rapid increase in *SOD1* mRNA expression. This is an important protective factor that limits acute peroxidative damage of skeletal muscles after eccentric exercise. Our results indicate that the CE program is most effective for up-regulating acute *SOD1* mRNA expression, followed by the IE program.

Our data also show that the main feature of the CE and IE programs was only the initial upregulation of acute *SOD* mRNA expression in the RFM, but no second upregulation. Expression of *SOD1* and *SOD2* mRNA at 0.5 h was higher after CE than OE or IE. Expression of *SOD1* mRNA at 0.5 h was higher after IE than OE. The results indicated that regular eccentric exercise could generate a large amount of *SOD* mRNA to facilitate adaptation of skeletal muscles to acute oxidative stress. The previous study reported that the 5′ promoters of SOD1 and SOD2 contain NF-κB binding sites [39], the activation of NF-κB pathway in rat skeletal muscle could elevate *SOD2* mRNA level and protein content [40], [43]. Several studies have shown that antioxidase activity in rat muscles can be induced by endurance exercise [8], [19], [44], which might result from increased ROS production [19], [20], [45], Powers et al. suggested that ROS could induce muscle adaptation in response to endurance exercise training by activating exercise-induced NF-κB and peroxisome proliferator-activated receptor γ coactivator-1α (PGC-1α) signaling pathways in skeletal muscle fibers [37]. It was suggested that mRNA expressions contributed to the adaptations of skeletal muscle gene with exercise [46], [47], and the all three kinds of *SOD* mRNA expressions in mouse tissues were regarded as one of index in organs adapting to oxidative stress during training [27]. We considered that regular eccentric exercise, especially CE program, was more beneficial to adaption of the skeletal muscles to acute oxidative damage after eccentric exercise.

In the present study, expression of *SOD1* mRNA significantly increased at 0.5 h in all 3 eccentric exercise groups and reached a peak at 4 days after OE. ROS may be critical to regulation of cell signaling pathways that promote gene expression [48], [49]. It was reported that *SOD1* mRNA expression significantly increased after acute contraction of skeletal muscles in Wistar rats [41], [42]. Our data showed that the mRNA expression of *SOD3* decreased 0.5 h after the CE and IE, and increased 4 days after OE, exhibiting the lowest fold increase. Hitomi et al. found that *SOD3* mRNA expression in mouse skeletal muscles could be increasingly induced after acute uphill treadmill exercise [28], which is inconsistent with our results. This discrepancy might be related to use of different laboratory animals and exercise models. We considered that the oxidative adaption ability of *SOD1* mRNA expression was the most important, followed by *SOD2* and *SOD3* mRNA expression, and the adaption of *SOD3* mRNA only occurs at 4 days after OE.

As we known, the greatest loss in peak and average torque/tension and lasting mechanical hyperalgesia were seen 12 hours following eccentric contractions. Zheng et al. [32] found that Leptin could enhance SOD1 and SOD2 activity, and stimulate mRNA expression of SOD2 (but not SOD1), and reduced MDA formation. Furthermore, SOD activation and induction of *SOD2* mRNA might be accompanied by ROS generation [32]. Our data show *SOD1*, *SOD2*, *SOD3* mRNA expression levels negatively correlated with MDA concentration at 12 h after CE. Imbalance of ROS production is induced by a dissociation between the local inflammatory response related to increased proinflammatory cytokine secretion (e.g., IL-1β, and TNF-α) and lack of systemic inflammatory response [50]–[53]. We therefore considered that *SOD* mRNA expression levels correlate with MDA concentration at the acute phase of CE, and are affected by exercise programs and time points. The present study found that OE is associated with increased *SOD1*, *SOD2*, and *SOD3* mRNA expression 96 h after exercise. Expression of *SOD1* mRNA was the highest, and *SOD2* mRNA expression levels negatively correlated with MDA concentration at 48 h and 96 h; whereas MDA concentration was same for OE at 48 h and control, and decreased at 96 h. This suggests that the ability of muscle to scavenge oxidative radicals is enhanced by OE at 96 h and indicates that the second upregulation of *SOD1*, *SOD2*, and *SOD3* mRNA expression occurs 96 h after OE.

Expression of *SOD* mRNA and *SOD* catalytic activity correlated with tissue metabolic rates. Specifically, they correlated with the oxidative capacity of different muscle fibers [20], [54]. However, the present study only analyzed *SOD* isoenzyme mRNA expression and MDA concentration in the RFM of SD rats, Therefore, the conclusions that can be drawn from this study may be limited. While the *SOD* mRNA expressions in tissues are one of index in organs adapting to oxidative stress after exercise [27]–[29], [55]. We considered that the RFM was the main muscle involved in lower limb exercise in SD rats; it was a mixed type dominated by IIa muscle fibers; the antioxidase activity of IIa muscle fibers was the highest; antioxidase activity in rat muscles is inducible through exercise [19], [56]. SOD isoenzyme could convert O_2_^−^ to hydrogen peroxide, which was one of the most important processes in the antioxidant defense system [28]. Therefore, we assessed the dynamic redox balance during lower limb exercise by studying changes in *SOD* isoenzyme mRNA expression and MDA concentration in the RFM.

In summary, our findings demonstrated that the mRNA expression of SOD isoenzyme and MDA concentration were significantly affected by different exercise programs over different time points in rat RFM tissue. The CE program most efficiently increased acute oxidative adaption capacity of skeletal muscles in *SOD1* and *SOD2* mRNA expression. Up-regulation of *SOD1* mRNA expression is a critical adaptive response by skeletal muscles to exercise at acute phase. Moreover, only OE induced the adaptive response of RFM in a delayed manner by elevating SOD expression. Such a finding may have significant implications for the formulation of athletic and physical training programs.
